# Contrastive learning-based pretraining improves representation and transferability of diabetic retinopathy classification models

**DOI:** 10.1038/s41598-023-33365-y

**Published:** 2023-04-13

**Authors:** Minhaj Nur Alam, Rikiya Yamashita, Vignav Ramesh, Tejas Prabhune, Jennifer I. Lim, R. V. P. Chan, Joelle Hallak, Theodore Leng, Daniel Rubin

**Affiliations:** 1 Department of Biomedical Data Science, Stanford University School of Medicine, 1265 Welch Road, Stanford, CA 94305 USA; 2 Department of Electrical and Computer Engineering, University of North Carolina at Charlotte, 9201 University City Boulevard, Charlotte, NC 28223 USA; 3 Department of Ophthalmology and Visual Sciences, University of Illinois at Chicago, Chicago, IL 60612 USA; 4 Department of Ophthalmology, Stanford University School of Medicine, Stanford, CA 94305 USA; 5 Department of Radiology, Stanford University School of Medicine, Stanford, CA 94305 USA

**Keywords:** Diagnostic markers, Biomedical engineering, Image processing, Diagnosis, Disease prevention, Medical imaging, Prognosis, Biomarkers, Translational research

## Abstract

Diabetic retinopathy (DR) is a major cause of vision impairment in diabetic patients worldwide. Due to its prevalence, early clinical diagnosis is essential to improve treatment management of DR patients. Despite recent demonstration of successful machine learning (ML) models for automated DR detection, there is a significant clinical need for robust models that can be trained with smaller cohorts of dataset and still perform with high diagnostic accuracy in independent clinical datasets (i.e., high model generalizability). Towards this need, we have developed a self-supervised contrastive learning (CL) based pipeline for classification of referable vs non-referable DR. Self-supervised CL based pretraining allows enhanced data representation, therefore, the development of robust and generalized deep learning (DL) models, even with small, labeled datasets. We have integrated a neural style transfer (NST) augmentation in the CL pipeline to produce models with better representations and initializations for the detection of DR in color fundus images. We compare our CL pretrained model performance with two state of the art baseline models pretrained with Imagenet weights. We further investigate the model performance with reduced labeled training data (down to 10 percent) to test the robustness of the model when trained with small, labeled datasets. The model is trained and validated on the EyePACS dataset and tested independently on clinical datasets from the University of Illinois, Chicago (UIC). Compared to baseline models, our CL pretrained FundusNet model had higher area under the receiver operating characteristics (ROC) curve (AUC) (CI) values (0.91 (0.898 to 0.930) vs 0.80 (0.783 to 0.820) and 0.83 (0.801 to 0.853) on UIC data). At 10 percent labeled training data, the FundusNet AUC was 0.81 (0.78 to 0.84) vs 0.58 (0.56 to 0.64) and 0.63 (0.60 to 0.66) in baseline models, when tested on the UIC dataset. CL based pretraining with NST significantly improves DL classification performance, helps the model generalize well (transferable from EyePACS to UIC data), and allows training with small, annotated datasets, therefore reducing ground truth annotation burden of the clinicians.

## Introduction

Diabetic retinopathy (DR) is a major ocular manifestation of diabetes. According to a World Health Organization (WHO) report, it is estimated that by the year 2040, the number of diabetic patients will reach 642 million. Nearly 40–45% patients with diabetes are prone to vision impairment due DR, making the global estimate of DR patients nearly 224 million. DR can be initially asymptomatic at its non-proliferative stage (NPDR), which is characterized by the presence of micro-aneurysms. If not treated in a timely fashion, it can progress to proliferative diabetic retinopathy (PDR), leading to irreversible vision loss and blindness. The American Academy of Ophthalmology (AAO) recommends that patients with the prevalent Type II diabetes should be screened every year after the initial diagnosis^1^. However, studies show that less than 50% diabetic patients follow through and get their yearly screening, the rate being even lower (15–20%) in rural areas^2,3^. Therefore, it is imperative to find an efficient way to improve the treatment management for DR and enable mass screening, early onset detection, and clinical diagnostics.

In recent years, researchers have successfully demonstrated machine learning (ML) and deep learning (DL) based algorithms for detecting diabetes^4^, DR diagnosis and referrals. Especially, DL methodologies have facilitated feature extraction and DR classification with high accuracy, sensitivity, and specificity^5–17^ using different imaging modalities such as fundus images, optical coherence tomography (OCT) and OCT angiography (OCTA) images. In general, such DL based DR classification pipelines require large, clean, diverse data, ground truth associated with the data, and a robust DL model (convolutional neural nets such as VGG16, ResNet, InceptionNet etc.). In case of referable vs non-referable DR classification, despite impressive performance showcased by these DL models, they often underperform in independent clinical test datasets. The reason for this is that the model often do not learn effective data representations that can lead to better generalization and model transferability. Furthermore, these models need to utilize larger, more diverse data from different sub-populations for better model training which increases the need for ground truth generation and data labeling—creating a large burden on clinicians. In this study, our goal is on reducing the burden of ground truth generation by developing more transferable DL models through a robust data representation learning framework.

To address this goal, we have developed a self-supervised contrastive learning (CL) based pipeline for classification of referable vs non-referable DR. Self-supervised models like CL help a DL model learn effective representation of the data without the need for large ground truth data^18,19^, the supervision is provided by the data itself. In such pipelines, a DL network is trained on a primary task (for representation learning, which requires no ground truth), and then the weights from that task are transferred to a secondary target task (i.e., classification, which requires smaller set of data and ground truth). For example, if a model is trained to solve an image puzzle, it does not need ground truth (the input image is the reference ground truth). But by learning to solve the puzzle, the model learns effective representation and the characteristic features from the image. This model’s weight can be then used for image classification task—yielding high classification performance with smaller data and ground truth^20^. In our case, while prior models on DR classification uses ‘ImageNet’ weights for transfer learning models^11,12,21–24^, our framework generates enhanced transfer learning weights that improves classification accuracy, even with smaller training data.

In this paper, our key novel contributions are following: (i) we present ‘FundusNet’—a novel CL based framework with neural style transfer (NST) based augmentation that achieves high classification performance; (ii) our novel NST based image augmentation technique effectively improves the representation learning capability of the CL network from fundus images as demonstrated by comparing to a state of the art lesion based CL model^25^. The model with FundusNet weights is independently evaluated on external clinical data, which achieves high sensitivity and specificity, when compared to three baseline models (two fully supervised models and one CL model); and iii) The CL-pretrained model also performed well even when the labelled dataset was reduced to 10% of its original size, suggesting the potential of CL to train models for DR diagnosis using small, labeled datasets.

## Results

### Study datasets

**Figure 1:**
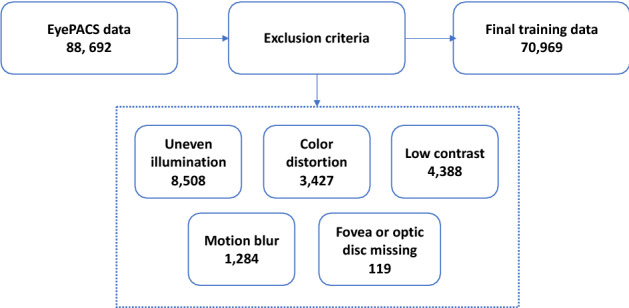
Data exclusion criteria for EyePACS training data.

This study used EyePACS dataset for the CL based pretraining and training the referable vs non-referable DR classifier. EyePACS is a public domain fundus dataset which contains 88,692 images from 44,346 individuals (both eyes, OD and OS), and the dataset is designed to be balanced across races and sex. After removing the fundus images that met the exclusion criteria (uneven illumination, color distortion, low contrast, motion blur, missing fovea or optic discs; Fig. 1), the final training dataset contains 70,969 fundus images, of which 57,722 are non-referable DR and 13,247 are referable DR. An independent testing dataset from UIC retina clinic is used for the target task of DR classification. This dataset contains 2500 images from 1250 patients (both eyes OD and OS). Among 1250 subjects (mean [SD] age, 53.37 [11.03]), 818 were male (65.44%) and 432 were female (34.56%). The UIC data was curated for this study from clinical DR patients at UIC retina clinic (not publicly available). The detailed demographic information of the subjects from UIC is in Table 1. There was no statistically significant difference in the distribution of age, sex, and hypertension between non-referable and referable DR groups (Analysis of Variance (ANOVA), P = 0.32, 0.18, 0.59 respectively).

**Table 1:** Demographics characteristics of non-referable and referable DR subjects from the testing dataset at UIC.

	Non-referable DR	Referable DR
Number of subjects	750	500
Sex (male/female)	458/292	360/140
Age (mean ± SD)	50.36 ± 10.64	56.37 ± 11.84
Age range	28–73	32–78
Duration of disease (years)	14.23 ± 10.22	19.32 ± 12.94
Diabetes type	Type II	Type II
Insulin dependent(Y/N)	117/633	389/111
HbA1C, %	6.6 ± 4.1	8.1 ± 3.2
HTN prevalence, %	69	81

*DR* diabetic retinopathy, *SD* standard deviation, *HbA1C* glycated hemoglobin, *HTN* hypertension.

### Framework for contrastive learning-based pretraining

**Figure 2:**
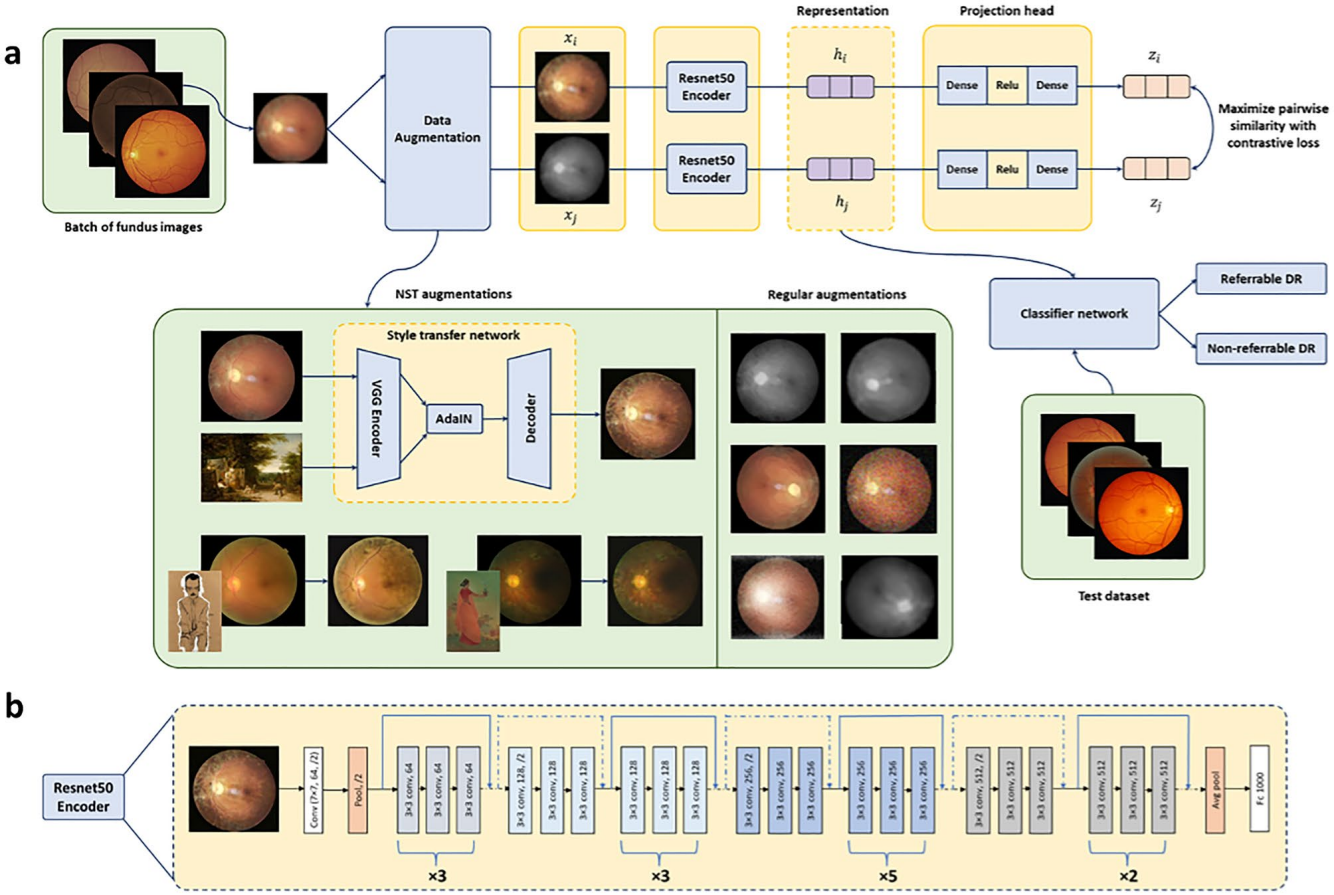
(**a**) A framework for contrastive learning based pretraining for referrable vs non-referrable diabetic retinopathy classification. NST denotes neural style transfer. The training utilizes the EyePACS dataset, whereas the test dataset comes from the UIC retinal clinic. The input to the contrastive learning framework is fundus images (x). xi and xj are augmented image pair from each x. The representations hi and hj are used as transfer learning weights (one-to-one for encoder layers) for the classifier network (Resnet50) after the contrastive learning pipeline is optimized, i.e., the contrastive loss has reached its minimum value. zi and zi refer to the final representation after the projection head. The AdaIN refers to adaptive instance normalization that allows real time style transfer, described in^26,27^. Regular augmentation refers to augmentations used in the original SimCLR paper^18^ (flipping, rotation, color distortion), (**b**) architecture of the ResNet50 encoder. *Conv* convolutional layer, *Pool* a pooling layer, *Avg pool* average pooling layer, *FC* fully connected convolutional layer.

Our FundusNet framework consists of two primary steps. First, we perform self-supervised pretraining on unlabeled fundus images from the training dataset using contrastive learning to learn visual representations. Once the model has been trained, the weights are transferred to a secondary classifier model for supervised fine-tuning on labeled fundus images. Figure 2 describes a summary of the framework.

To teach our model visual representations effectively, we adopt and modify the SimCLR framework^18^, which is a recently proposed self-supervised approach that relies on contrastive learning. In this method, the model learns representations by maximizing the agreement between two differently augmented versions of the same data using a contrastive loss (more details on contrastive loss is provided in “Material and methods” section). This contrastive learning framework (Fig. 2a) attempts to teach the model to distinguish between similar and dissimilar images. Given a random sample of fundus images, the FundusNet framework takes in each image x, augments them twice, creating two versions of the input image x_i_ and x_j_. The two images are encoded via a ResNet50 network (Fig. 2b), generating two encoded representations h_i_ and h_j_. These two representations are then transformed via a non-linear multi-layer perceptron (MLP) projection head, yielding two final representations, z_i_ and z_i_, which are used to calculate the contrastive loss. Based on the loss on each augmented pairs generated from a batch of input images, the encoder and projection head representations improve over time and the representations obtained place similar images closer in the representation space. The CL framework contains a Resnet50 encoder (containing convolutional neural network and pooling layers with skip connections) with a projection head (dense and Relu layers) that maps the representation. The batch size of the CL pretraining pipeline has been demonstrated to have significant effect on the model pretraining^18,19,28^ and therefore, the performance of the target model. To test this, we trained our FundusNet model for bath sizes 32 through 4096 (step size 32) in the CL pretraining step. The model is trained for 100 epochs or until the loss function saturates.

### Improving representation learning through neural style transfer (NST)

One of the key findings from CL based self-supervised pretraining is that augmentation and transformation are key to better representation learning. As we adopted and modified the SimCLR framework, which was originally used for classification of natural images, we found that regular image augmentation techniques such as flipping, rotating etc. did not generate a good representation (Z_i_ and Z_i_ in Fig. 2a) from fundus images. A study by Geirhos et.al.^27^ demonstrated that CNNs used in computer vision tasks are often biased towards texture, compared to global shape features that are primarily used by humans for distinguishing classes. Increasing shape bias by randomizing texture environments can be a useful way to improve accuracy and generalizability of a CNN model. NST manipulates the low-level texture representation of an image (style) but preserves the semantic content. NST has been previously demonstrated to improve robustness to domain shift in CNNs for computer vision tasks^29,30^. In our study, we integrated an NST-based augmentation technique into the CL pipeline, based on convolutional style transfer from non-medical style sources (i.e., art, painting etc.). The NST replaces the style of the fundus images (primarily texture, color and contrast) with the randomly selected non-medical images. However, it preserves the semantic contents (global objects, shapes like microaneurysm, vasculature etc.) of the image required for better disease detection. The NST convolution methodology was adopted from AdaIn style transfer^26,27^. The style source was artistic paintings from Kaggle’s ‘Painter by Numbers’ dataset (79,433 paintings), downloaded via https://www.kaggle.com/c/painter-by-numbers. In the CL pretraining, the NST based augmentation was combined with the regular augmentation techniques such as rotation, flipping, color distortion, crops with resize, and gaussian blur. A higher probability (70%) of augmentation through NST was defined in the pretraining protocol. To compare the performance improvement of detecting referable DR due to integration of NST into our pipeline, we also trained two baseline CL frameworks: first one with just the original SimCLR augmentations^18^ and a second state of the art lesion based CL model that utilized lesion patches instead of the whole fundus image (with original SimCLR augmentations).

### Referable vs non-referable DR classification training schemes

Using the weights of the pretrained network as initializations, we trained an end-to-end supervised model for a downstream DR classification task (referable vs non-referable DR). We trained a ResNet50 encoder network with standard cross-entropy loss, a batch size of 256, ADAM optimizer and random augmentations (gaussian blurring, resizing, rotations, flipping, and color distortions). The transfer learning weights were encoder to encoder (one-to-one; Fig. 2), i.e., the h representations from the CL network (before the projection head) were transferred to a ResNet50 encoder. To compare our FundusNet results, we also trained two separate fully supervised baseline models (ResNet50 and InceptionV3 encoder networks, both initiated with Imagenet weights). Both the baseline models are based on based on DL models in literature that have achieved state of the art diagnostic accuracy in detecting referable DR^17,24^. Standard hyperparameter search (learning rate (logarithmic grid search between 10^–6^ and 10^–2^), optimizer (ADAM, SGD), batch size (32, 64, 128, 256)) and training protocols were maintained for the FundusNet and both baseline networks.

To further investigate whether the CL pretrained model performs well with smaller training data (and ground truth), we reduced the training dataset gradually from 100 to 10% (10% step size) and conducted the downstream classification training for both the CL and Imagenet pretrained baseline models. After identifying the best hyperparameters and fine tuning the models for each experiment, we chose the model that had the best performance on validation dataset (fivefold cross validation). The final optimal models were tested on an independent testing dataset from UIC. In terms of encoder networks, we compared three types of encoder networks in our experiment (VGG, ResNet, and Inception architectures).

### FundusNet performance on real-life clinical test data

The FundusNet model pretrained with style transfer augmentation achieved an average area under the receiver operating characteristics (ROC) curve (AUC) of 0.91 on the independent test dataset from UIC, outperforming the state-of-the-art supervised baseline models (ResNet50 and InceptionV3) trained with Imagenet weights^17^ (AUCs of 0.80 and 0.83, respectively), and the CL baseline models trained with SimCLR and lesion based framework (AUCs of 0.83 and 0.86 respectively) (Tables 2, 3). The significant performance difference in the testing test compared to the baseline model indicates that the FundusNet model generalized better through our pretraining framework due to enhance representation learning enabled by NST. The NST augmentation that was designed specifically for learning global geometric features, allowed learning of more discriminative visual representations of retinal pathologies, improving the overall classification performance compared to CL models pretrained with generic augmentation techniques.

**Table 2:** Classification performance of FundusNet framework for referable vs non-referable DR compared to fully supervised baseline models.

Models	AUC (95% CI) cross-validated on EyePACS dataset	AUC (95% CI) on test dataset	P-value (vs FundusNet)	Sensitivity (95% CI)	P-value (vs FundusNet)	Specificity (95% CI)	P-value (vs FundusNet)
FundusNet	0.96 (0.938–0.972)	0.91 (0.898–0.930)	Ref	0.90 (0.895–0.917)	Ref	0.85 (0.830–0.862)	Ref
Baseline1 (ResNet50)	0.94 (0.919–0.953)	0.80 (0.783–0.820)	P < 0.001	0.81 (0.793–0.834)	P < 0.001	0.74 (0.731–0.758)	P < 0.005
Baseline2 (InceptionV3)	0.92 (0.905–0.961)	0.83 (0.801–0.853)	P < 0.001	0.84 (0.822–0.848)	P < 0.001	0.79(0.786–0.819)	P < 0.05

P value from measuring statistical significance using DeLong’s test for comparing pairwise AUCs.

*DR* diabetic retinopathy, *CI* confidence interval, *AUC* area under the ROC curve, *Ref* reference.

**Table 3:** Evaluation of model performance on test set based on augmentation and contrastive learning techniques compared to the state of the art.

Method	AUC (95% CI)	
	100% data	10% data
SimCLR^18^	0.83 (0.80–0.85)	0.71 (0.66–0.73)
Lesion based CL (confidence threshold 0.7)^25^	0.86 (0.81–0.87)	0.75 (0.71–0.79)
Lesion based CL (confidence threshold 0.8)^25^	0.87 (0.84–0.89)	0.75 (0.74–0.78)
FundusNet (final proposed model)	0.91 (0.898–0.930)	0.81 (0.77–0.84)

Confidence threshold refers to the threshold set in^25^ to reduce un confident prediction of patches from fundus images.

*DR* diabetic retinopathy, *CI* confidence interval, *AUC* area under the ROC curve, *CL* contrastive learning.

**Figure 3:**
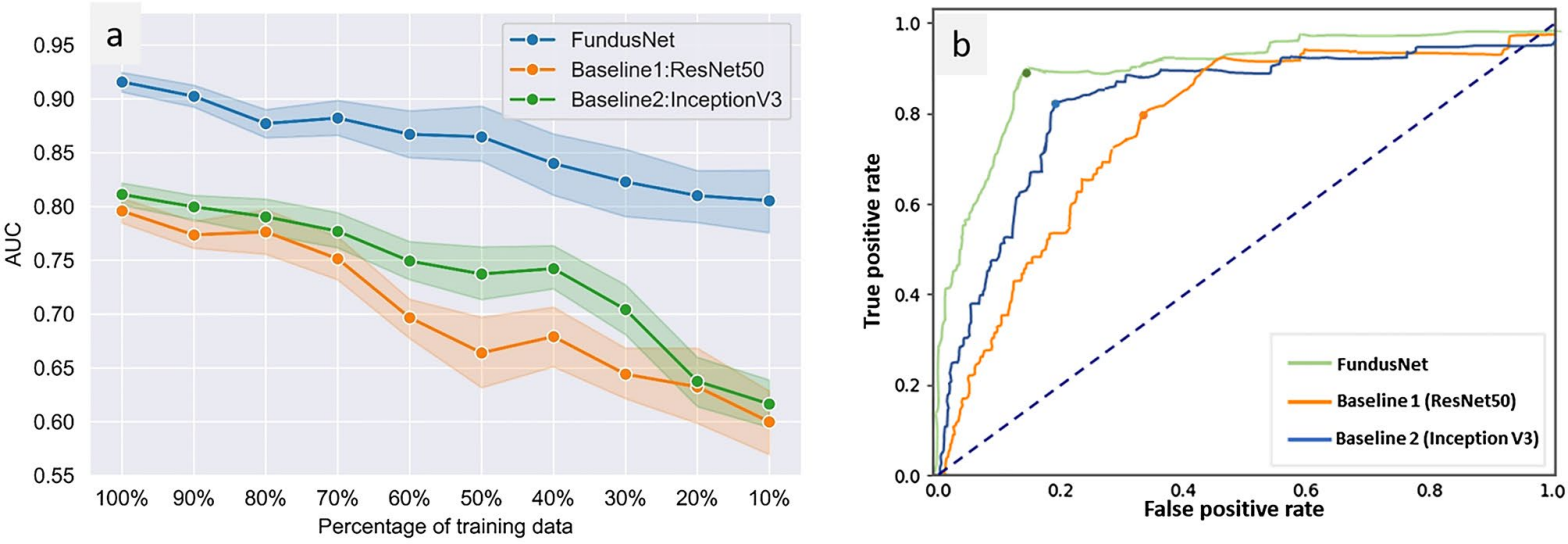
(**a**) AUC for referrable vs non-referrable DR classification tested on independent test data from UIC for FundusNet vs two baseline models with varied percentages of training data, (**b**) ROC curve for FundusNet and two baseline models (test set).

To investigate the label-efficiency of the FundusNet model, we trained our model on different fractions of the labeled training data and tested each resulting model on the test dataset. We compared this to the performance of the baseline models. Fine-tuning experiments were conducted on five-folds training data and the results were averaged. Figure 3a shows how the performance varies using the different label fractions for both the FundusNet and baseline supervised models on testing dataset. We observe that the CL pretrained FundusNet model retains AUC performance even when the labels are reduced up to 10%, whereas there is significantly smaller performance of the baseline models. When reducing the amount of training data from 100 to 10% of the data, the AUC for FundusNet drops from 0.91 to 0.81 when tested on UIC data, whereas the drop is larger for the baseline models (0 0.80 to 0.58 for the ResNet50 and 0.81 to 0.63 for the InceptionV3 model). Importantly, the FundusNet model is able to match the performance of the baseline models using only 10% labeled data when tested on independent test data from UIC (FundusNet AUC 0.81 when trained with 10% labelled data vs 0.80 and 0.81, respectively, for baseline models trained with 100% labelled data).

In the experiment to evaluate the optimal batch size for CL pretraining, we observed that CL frameworks learned better image representations when there were higher number of negative examples in a batch (i.e., augmented image pairs generated from other images in a batch), therefore, higher batch size yielded better performance (Table 4; AUC of 0.77 for batch size 32 vs 0.91 for batch size 2048 on test dataset). However, high batch size also means the need for larger compute resources. We observed that at batch size 4096, the AUC did not improve significantly, so the optimum batch size was chosen as 2048. In terms of encoder networks, compared to VGG and Inception architectures, ResNet50 provided the best classification performance in both validation and test dataset (Table 5).

**Table 4:** Effect of batch size on FundusNet performance on detecting referable DR.

Batch size	AUC (SD)—test dataset	P value (vs 32 batch size)	P value (vs 2048 batch size)
32	0.77 (0.039)	REF	< 0.001*
64	0.78 (0.058)	0.18	< 0.001*
128	0.78 (0.045)	0.16	< 0.001*
256	0.80 (0.077)	0.06	< 0.001*
512	0.86 (0.082)	0.02*	< 0.001*
1024	0.87 (0.034)	< 0.01*	< 0.01*
2048	0.91 (0.014)	< 0.001*	REF
4096	0.91 (0.011)	< 0.001*	0.08

*AUC* area under the ROC curve, *SD* standard deviation, *Ref* reference.

*Significant difference; P value from measuring statistical significance using DeLong’s test for comparing pairwise AUC values among the batch size (all vs batch size 32 in third column, and all vs batch size 2048 in fourth column). This pairwise comparison was done to show whether the AUCs coming from experiments with all the batch sizes are significantly different that AUCs coming from experiments with batch size 32 (initial batch size) and 2048 (optimal batch size).

**Table 5:** Classification performance using different encoder networks.

Encoder architecture	AUC (SD) on test dataset
Resnet50	0.91 (0.01)
Resnet152	0.90 (0.032)
Vgg16	0.82 (0.044)
InceptionV2	0.89 (0.019)
InceptionV3	0.90 (0.064)

*DR* diabetic retinopathy, *CI* confidence interval, *AUC* area under the ROC curve, *Ref* reference.

**Figure 4:**
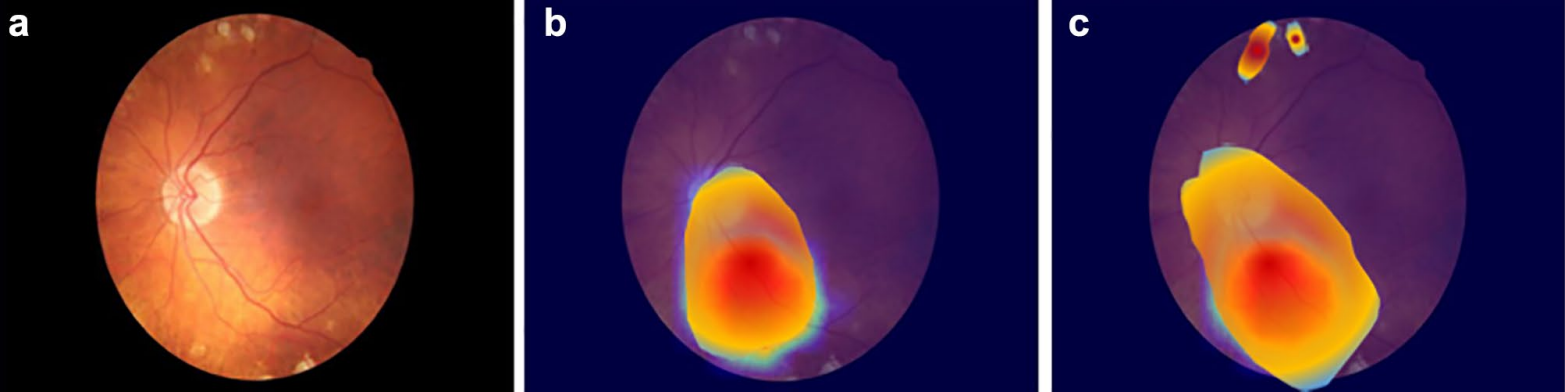
(**a**) Sample PDR fundus image, (**b**) saliency map from ResNet50 model with ImageNet weight, (**c**) saliency map from ResNet50 model with FundusNet weights.

We have also generated saliency maps using GradCAM^31^ to visualize regions in the retina that were most likely used by our models to predict DR stages. Two sample saliency maps from a PDR subject are shown in Fig. 4, which are generated from a ResNet50 model (pretrained with ImageNet and FundusNet weights). The hotter colors like red and oranges are indicative of regions which potentially contribute more towards the predicted output. We can observe that, the saliency map near the optic disc is similar in both models, however, ResNet50 with FundusNet weights looks at additional regions on the top of the fundus images, that have some physiological markers.

## Discussion

In this paper, we present a self-supervised CL based pipeline, FundusNet, for improving the performance, of referable vs non-referable DR classification over previously published baseline models^17,24^. CL improves representation and transferability of DR classification models. Our key contributions are: (i) the novel NST based CL pretraining method significantly outperformed the baseline models pretrained with Imagenet weights; (ii) we proposed a novel NST based augmentation within the CL framework that enhances the capability of the model to learn fundus data representation for robust classification performance. The augmentation outperforms generic SimCLR and state of the art lesion-based CL framework in terms of pretraining a model for better transferability; and iii) the CL pretrained models performed well, even when we reduced the amount of training data by 90%. The FundusNet network with CL pretraining shows potential for integrating self-supervision in deep learning frameworks for developing robust diagnostic models with reduced amount of data and associated ground truth. Previous models for the diagnosis of DR used Imagenet weights to initiate their models^11,12,21–24^. While transfer learning through Imagenet can be useful, recent work has shown that the role of Imagenet weights for medical imaging tasks can be limited^32,33^ since it was developed and validated on natural images, and pretraining on in-domain medical image data (i.e., FundusNet weights) can be more effective^33^ compared to Imagenet. Our CL framework provides an enhanced transfer learning weights that can be used in other applications pertaining retinal images and disease diagnosis.

The FundusNet model achieves high sensitivity and specificity in referable vs non-referable DR classification (Table 2) and performed significantly better than the supervised baseline models (ResNet50 and InceptionV3^17,24^) on the independent test dataset (AUC of 0.91 vs 0.80 and 0.81 respectively) and on EyePACS fivefold validation data (AUC of 0.95 vs 0.92 and 0.94 respectively), suggesting that the CL model generalized better. Several previous studies have demonstrated great success to detect referable DR using deep learning approaches. Gulshan et al. developed a model with 128,175 images and validated the model on 9963 fundus images high level of performance for classifying referable DR (AUC = 0.99)^8^. Ting et al. trained their deep learning model using 73,370 images and reported excellent results for referable vs non referable DR (AUC of 0.936)^15^. Li et al. trained their deep learning system on 71,043 images and validated them in a real-world dataset of 35,201 images (AUC of 0.955 for vision-threatening DR)^24^. To achieve such excellent diagnostic accuracy, these works used a large diverse training dataset (ranging from 70,000 to 200,000 images) with class labels created by multiple clinicians. Our CL framework could potentially reduce the need for large training datasets. On the independent test data from UIC, the FundusNet model matched the AUC of the two baseline models with only 10% of labeled training data (Fig. 3).

In our study, we not only adapted contrastive learning into our training pipeline, we also further modified the pipeline with a novel NST based augmentation. NST is an image style transformation algorithm that manipulates the low-level texture representation of an image while preserving the semantic content. NST has been previously demonstrated to improve robustness to domain shift in CNNs for computer vision tasks^20^, and we have adopted it into our FundusNet framework. This is a unique application of style transfer from medically irrelevant artistic images in ophthalmic diagnostics. The NST augmentation facilitated learning discriminative visual representations of retinal pathologies by encouraging the model to learn global shape based structural characteristics of vasculature and other pathologies in the retina. NST works well as an augmentation method because the style transfer can introduce in a wide variety of textures when training classifiers using medical images (which often have more uniform color and texture distribution), that can improve classifier performance. Therefore, compared to conventional augmentations (such as the ones used in the original SimCLR^18^), the model generalizes better when augmented with NST using texture-rich artistic images (AUC of 0.91 vs 0.83 when FundusNet used NST vs did not used NST on top of regular augmentations). We compare the NST based framework with another recent work that proposed a lesion based CL framework^25^ for DR classification, where the model input is segmented lesions, rather than the whole fundus images. The augmentation technique used in this work is similar to that of original SimCLR paper. We implemented this framework for comparison and observed that the lesion-based CL pretraining did not provide significant performance improvement compared to SimCLR framework (Table 3). Possible reason is that, this method requires to employ a patch detection model prior to CL pretraining, that does not generalize very well to new datasets (i.e. EyePacs or UIC data) and can lead to a very large number of patches that do not have any pathologies. Furthermore, the generic augmentation techniques do not cater well for retinal image representation learning. Our proposed framework with NST can identify more global features that can be useful for DR classification. When compared, the NST model outperformed the lesion-based CL framework (AUCs of 0.91 vs 0. 84 for 100% labelled data and 0.81 vs 0.75 for 10% labelled data). Furthermore, our NST model does not require any additional step for patch identification that can add processing time and complicate both training and evaluation of models when used on new external datasets.

Our study has several limitations. First, we did not automatically optimize the NST hyperparameters. The use of NST as an augmentation technique is new and requires further investigation to optimize the style coefficients. Another limitation of our approach is that a large batch size is required for training of the CL model. Self-supervised frameworks like SimCLR and MoCo reported the need for larger batch size^18,19,28^ because CL training requires a large number of negative samples in a batch to calculate contrastive loss efficiently. This can impair computational efficiency. Furthermore, our model was tested on only one external clinical site at UIC, which may not be sufficiently representative of a broader population. Further validation of the developed FundusNet framework is required in future studies.

In conclusion, we have introduced a self-supervised CL based framework for referable vs non-referable DR classification that includes NST augmentation for learning effective pathological and visual representations from retinal fundus images. Our experiments demonstrate that our CL based pretraining yields significant improvements of DR classification compared with the baseline models in independent testing data (AUC (CI) values of 0.91 (0.898 to 0.930) in FundusNet vs 0.80 (0.783 to 0.820) in ResNet50 and 0.83 (0.801 to 0.853) in InceptionV3 baseline models). Importantly, our CL model is able to be trained using only 10% of the training data that could reduce the annotation burden for clinicians in producing training datasets. The FundusNet model is able to match the performance of the baseline models using only 10% labeled data when tested on independent test data from UIC (FundusNet AUC 0.81 when trained with 10% labelled data vs 0.80 and 0.81, respectively, for baseline models trained with 100% labelled data). Ultimately, our CL method could be useful for developing classification models for clinical use when validated sufficiently.

## Materials and methods

### Study design and participants

This study was approved by the institutional review board (IRB) of Stanford University and the University of Illinois at Chicago (UIC) and was in compliance with the ethical standards stated in the declaration of Helsinki. Informed consent was obtained from all subjects to use the de-identified data in this research. This multi-center cross-sectional study was primarily conducted at Stanford University School of Medicine. The testing data from UIC was shared in encrypted cloud drive with researchers at Stanford. For training and developing the CL based pretraining and referable vs non-referable DR classifier, we used the EyePACS dataset from Kaggle (88,692 fundus photographs, EyePACS, California). The final DR classifier model was tested on an independent dataset from UIC. The training data from EyePACS contained retinal fundus photographs from patients with varying degrees of severity of DR. The dataset had 57,722 non-referable and 13,247 referable DR images with varying resolutions from 433 × 289 up to 5184 × 3456 pixels. Images from the dataset are already labeled with stages of DR (0: no DR, 1: mild, 2: moderate, 3: severe non-proliferative DR (NPDR), and 4: proliferative DR (PDR)), following the diagnostic criteria for DR. For our project, we define referable DR as data which have labels of moderate NPDR and above (label ≥ 2). We excluded images that satisfied our exclusion criteria (uneven illumination, color distortion, low contrast, motion blur, missing fovea or optic discs; Fig. 1). An automated image quality assessment algorithm was used to identify the images that fit the exclusion criteria^34,35^. An ophthalmologist then manually verified the exclusion criteria and the excluded images. One image could satisfy multiple exclusion criteria, but the primary criteria was identified from the automated assessment algorithm (criteria with higher probability) and then confirmed by the ophthalmologist. The testing dataset from UIC contained 2500 fundus photographs from patients with DR, recruited from the UIC retina clinic (1000 referable and 1500 non-referable DR). This was retrospective data of type II diabetes patients who underwent retinal imaging at the clinic. The patients are thus representative of a university population of diabetic patients who require imaging for management of diabetic macular edema and DR. Images of both eyes were taken. Subjects with macular edema, previous history of eye diseases, and vitreous surgery were excluded from the study. The patients were classified by severity of DR according to the Early Treatment Diabetic Retinopathy Study staging system, which was then converted to class labels of no DR, mild, moderate, and severe NPDR, and PDR. The grading was done by retina specialist on dilated patients who were examined using a slit-lamp fundus lens, and technicians did not contribute to the grading of the patients. All patients in this study provided written informed consent and did not receive any compensation or incentives to participate.

### Contrastive learning-based pre-training

The CL framework learns representations by maximizing the agreement between two different augmented encodings (z_i_ and z_j_ in Fig. 2) of the same input data via contrastive loss within a hidden representation of neural nets^19^. The contrastive loss between a pair of positive augmented examples is defined as^36^:

1 $${loss}_{i,j}=-log\frac{\mathrm{exp}(sim({z}_{i}/{z}_{j})/\tau )}{{\sum }_{k=1}^{2N}{1}_{[k\ne 1]}exp(sim({z}_{i}/{z}_{k})/\tau )}$$

Here, sim(.) is a cosine similarity function computed on ${z}_{i}$; ${1}_{[k\ne 1]}\in$^5^ is a function evaluating up to 1 if and only if $k$ is not equal to $i$; $\tau$ is an adjustable temperature parameter that can scale the inputs and widen the range [−1, 1] of cosine similarity, and N is the batch size. Upon calculating the cosine similarity, a SoftMax function is applied to get the probability of the two pairs being similar. This SoftMax calculation is equivalent to getting the probability of the augmented image pairs being the most similar to each other. Here, all remaining images in the batch are sampled as a dissimilar image (negative pair). The loss is calculated for a pair by taking the negative of the log of this SoftMax calculation. In the pipeline, the final loss is computed across all the positive pairs in a batch. Based on the loss, the encoder and projection head representations improve over time and the representations obtained place similar images closer in the space. Once the CL model is trained on the contrastive learning task, it can be used for transfer learning.

The CL pre-training is conducted for a batch size of 32 through 4096. The large batch size can be unstable when using standard stochastic gradient descent with linear learning rate scaling^37^. To stabilize the CL pre-training, a LARS optimizer^38^ is used for all batch sizes. We train our model with Cloud TPUs, using up to 12 v2 cores depending on the batch size. With 12 cloud TPUs, it takes around 18 h to pre-train a ResNet-50 encoder with batch size of 2048 for 100 epochs.

In default setting of the CL pre-training, a ResNet50 base encoder along with 2-layer multi-layer perception (MLP) projection head were used to project the Z representations to a 128-dimensional embedded space. The loss from Eq. (1) was optimized with the LARS optimizer with a learning rate of 0.3 with a weight decay of 1e−5. The batch size with best performance was 2048 with 100 epochs. The pre-training experiments were conducted with or without initializing Imagenet weights. The augmentations with the style transfer comprised random horizontal and vertical flips, Gaussian blur, partial rotation up to 30°, and random color augmentation and jittering (strength = 0.5). All the images were resized to 224 × 224.

### Referable DR vs non-referable DR classification

For the task of referable vs non-referable DR classification, a ResNet50 network was trained with a batch size of 256 (image size 224 × 224), standard cross-entropy loss optimized with the ADAM optimizer. An on-the-fly random data augmentation was conducted (rotations (up to 30°), horizontal flipping, and color distortions). We experimented with the learning rate and weight decay (logarithmic grid search between 10^–6^ and 10^–2^ and 10^–5^ and 10^–3^ respectively). For the Imagenet supervised model as baseline, similar methods for data augmentation and hyper-parameter search were used.

### Suggested configurations

For the CL pre-training, at the default setting, at least a 4 core TPU core is suggested. Otherwise, the memory will be limited for CL pre-training. Post-CL pre-training, any desktop or laptop computer with × 86 compatible CPU, 8 GB or more of free disk space, and at least 8 GB memory are suggested for training and testing the referrable vs non-referrable DR classification framework with FundusNet weights. A computer with a GPU would make the training time significantly lower (12 h in a CPU vs 3 h in a GPU).

### Augmentation with neural style transfer

The stylized version of the fundus dataset is generated by applying AdaIn style transfer^26,27^. The AdaIn style transfer takes an input image (fundus) and a random artistic style image as inputs, then generates an output image that retains the content of the input image and the style of the artistic image. Within the framework, both the input and style images are encoded into the feature space and both the feature maps are fed into the AdaIN layer which aligns the mean and variance of the input feature maps to those of the style feature maps, therefore, producing the target feature maps. A decoder is then used to generate the stylized output image from the target feature maps. The AdaIn style transfer is chosen since it is capable of transferring the random styles in real time. Each of the fundus image was stylized through the AdaIn with a stylization coefficient of 1.0. The artistic style images were used from the ‘Painter by Numbers’ dataset (79,433 paintings), available in *Kaggle*. During the NST augmentation, the input images were resized to 512 × 512, and the style source images were resized to 128 × 128 to maintain the shape and global characteristics of the input image during stylization. The NST augmented images were synthesized in advance and called on the fly during CL pre-training. As discussed in the discussion section, the NST augmentation itself on the fly is computationally very expensive.

### Statistical analysis

Our primary metrics to evaluate the model performance was the area under the curve (AUC) with 95% confidence intervals (CI). For each experiment, sensitivity and specificity of the CNN classifiers were also computed across probability thresholds to plot the receiver operating characteristic (ROC) curves and calculate AUC. For the classification thresholds for generating ROC curve and concurrent analyses, we used Youden’s index. The optimal cut-off to get the best sensitivity and specificity results (Table 2) was based on Youden’s J = max(sensitivity + specificity − 1), also shown in Fig. 3b.

### Ethics

This study was approved by the institutional review board (IRB) of Stanford University and the University of Illinois at Chicago (UIC) and was in compliance with the ethical standards stated in the declaration of Helsinki. Informed consent was obtained from all subjects to use the de-identified data in this research.

## Data Availability

The validation dataset from EyePACS is available at https://www.kaggle.com/c/diabetic-retinopathy-detection/data. The test dataset from UIC can be made available upon request to the corresponding author Minhaj Alam (minhajnur.alam@gmail.com).

## Code availability

The code is open-source. All the code and FundusNet weights are available in https://github.com/malam8/FundusNet-DR.
